# ‘I feel many of my reflections are forced’: International medical graduates’ perspectives on reflection in UK general practice training: a mixed methods qualitative study

**DOI:** 10.3399/BJGPO.2023.0210

**Published:** 2024-10-10

**Authors:** Laura Jayne Emery, Ben Jackson, Caroline Mitchell

**Affiliations:** 1 Academic Unit of Primary Care, The University of Sheffield Faculty of Medicine Dentistry and Health, Sheffield, United Kingdom

**Keywords:** reflection, postgraduate education, international graduates, learning, education, qualitative research

## Abstract

**Background:**

UK general practice training requires trainees to evidence clinical competencies through reflective writing entries in online portfolios. Trainees who complete their medical degree in the UK experience reflection as an undergraduate, whereas 80% of international medical graduates (IMGs) have no previous experience of reflection.

**Aim:**

To explore IMGs’ perspectives on the positive and negative aspects of reflection in the context of postgraduate GP training.

**Design & setting:**

A mixed-methods qualitative study undertaken in the UK. Qualitative ‘free-text’ survey data obtained in 2021 were analysed. The themes were further explored by semi-structured interviews conducted in 2022–2023.

**Method:**

Participants were IMGs with experience of the UK GP training scheme. Verbatim open-question survey data underwent content analysis. Broad themes identified were used to develop the interview topic guide. A geographically dispersed, purposive sample of participants were recruited for semi-structured interviews. Interview and survey data were then analysed thematically.

**Results:**

In total, 433 participant datasets are included: 422 of 485 responses to a UK-wide survey, including open questions, and 11 interview transcripts. IMGs considered reflection to provide an effective approach for learning, an opportunity for self-assessment and professional development, and a means of developing self-awareness. Concerns were expressed about how time-consuming recording reflection is, how its mandated aspect makes it forced, and fears regarding the medico-legal consequences of reflective writing.

**Conclusion:**

Despite a lack of previous experience in reflection, most IMGs showed an understanding of the benefits of reflection in GP training. However, the challenges of reflection must be addressed, to avoid devaluing reflection for clinical learning.

## How this fits in

Previous research shows that 80% of international medical graduates (IMGs) have not experienced reflection prior to entering GP training. This research reports that, despite this, IMGs value reflection as an effective tool for professional development. However, concerns over time taken to write reflections, reflections being mandated and therefore feeling forced, and risks of medico-legal complications, threaten to devalue reflection. Reducing numbers of written reflections, adopting individualised approaches to reflection, and using established tools for improving depth of reflection will help address some of these issues.

## Introduction

In common with other specialty training programmes, written reflection within online portfolios continues to be integral to UK general practice training, as a means to evidence achievement of General Medical Council (GMC)-appointed competencies in order to progress through GP training and achieve mandatory Royal College of General Practitioners (RCGP) membership.^1^ The relationship between reflection and assessment is problematic. Previous studies suggest that when written reflection is used for the purposes of assessment, this changes the nature of what is written; reflection becomes a performance tailored to what students think their teachers want to hear.^2–4^ While the benefits of reflection are often extolled, there is less published information about these negative aspects of reflection.^5^

Student reflection is a GMC requirement for UK undergraduate medical curricula,^6^ but reflection is less common in international medical schools where a more didactic approach to education is favoured.^7^ Understanding the differences in educational experience is important as more than 40% of GP trainees in the UK are international medical graduates (IMGs).^8^ A survey of IMGs working in GP training in the UK showed that up to 80% have not experienced reflection in any form during their undergraduate training.^9^ It has been suggested that this lack of familiarity with reflection puts IMGs at risk of the medico-legal consequences of reflection.^10^

Building on a previous survey of IMGs' experiences of reflection,^9^ this study examines the qualitative survey data, triangulated with data from subsequent semi-structured interviews to gain greater understanding of IMG perspectives of positive and negative aspects of reflection in postgraduate GP training.

## Method

### Data gathering

Qualitative survey data, exploring IMGs’ perspectives on their experiences of reflection in postgraduate training (March–April 2021), were subjected to an initial content analysis focusing on the positive and negative aspects of reflection. Open questions about the best and worst aspects of reflection were analysed individually and as a complete dataset.

Themes identified were used to inform the content of a semi-structured interview topic guide, which was co-produced by the research team and members of a stakeholder group comprising GP trainees and qualified GPs with experience of working and training abroad. The topic guide allowed a more nuanced exploration of perspectives of reflection and was subject to iterative review as interviews progressed.

Interviews were conducted over the Google Meet platform by the lead researcher (LE) between June 2022 and January 2023. Written consent was obtained before the interview and confirmed before starting. Audio data were uploaded to secure university storage immediately following completion and transcribed by a professional transcription service. Participant identifiable information was removed from the final transcript.

### Recruitment of participants

**Figure 1.**
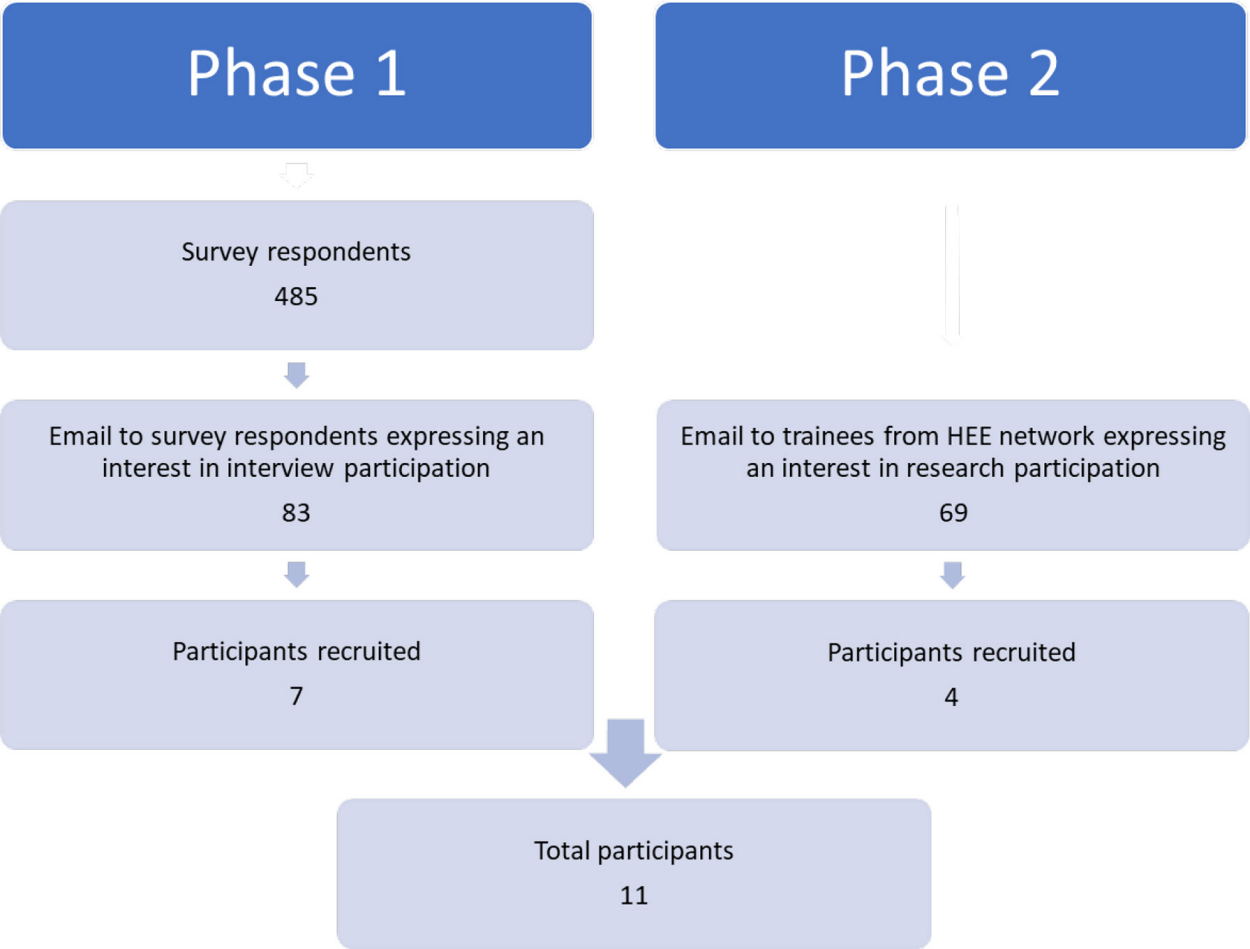
Phases of recruitment. HEE = Health Education England

Recruitment of interview participants was conducted in two phases (Figure 1). First phase recruitment was from a pool of survey responders who expressed an interest in taking part in follow-up interviews. Second phase recruitment was purposive in order to achieve a maximum variety sample in terms of sex and country of primary medical qualification. This was facilitated through links with Health Education Yorkshire and the Humber. Potential participants were sent a brief introductory email. Responders were then sent the participant information sheet and interviews were arranged.

### Data analysis

As expected, interviews produced richer, more detailed data than the dichotomous best or worst questions from the survey. The decision was therefore made to return to the data, and undertake thematic analysis of free-text survey responses to questions on the best and worst aspects of reflection (422 of 485 participants) alongside the interview transcripts (11 participants).^11^ Initial codes were developed and applied to both datasets, and higher-level themes identified through an iterative process of constant comparison. Independent verification of themes identified from the qualitative data was undertaken by BJ and CM. The final thematic framework, which integrated survey qualitative data analysis with the key themes from interview data analysis, was developed and subjected to critical interpretive challenge within regular research meetings (LE, BJ, CM).

## Results

In total, 485 IMG trainees from ethnically diverse groups participated in the online survey and 422 provided free-text survey responses. Although not compulsory, at least one question asking for opinions on best and worst things about reflection was completed by the majority of participants. Eleven trainees were recruited to interview (Figure 1). Interview duration was between 25 and 50 minutes. Demographic data are presented in Table 1.

**Table 1.** Participant demographics

	Interview participants (*n* = 11)	Survey participants (*n* = 422)
**Sex**		
Male	5 (45.5%)	188 (44.5%)
Female	6 (54.5%)	230 (54.5%)
Prefer not to say	0 (0%)	4 (1.0%)
**Ethnicity**		
Arab	1 (9.1%)	34 (8.1%)
Asian	5 (45.5%)	161 (38.2%)
Black	5 (45.5%)	153 (36.3%)
Mixed	0	11 (2.6%)
White	0	41 (9.7%)
Other	0	15 (3.6%)
Prefer not to say	0	7 (1.7%)
**Area of the world in which primary medical qualification achieved**		
Africa	3 (27.3%)	158 (37.4%)
EEA Europe	0 (0%)	52 (12.3%)
Non-EEA Europe	1 (9.1%)	10 (2.4%)
Middle East	2 (18.2%)	41 (9.7%)
Oceana	0 (0%)	2 (0.5%)
Rest of Asia	2 (18.2%)	19 (4.5%)
South Asia	2 (18.2%)	127 (30.1%)
South, Central, Latin America, and Caribbean	1 (9.1%)	13 (3.1%)
**Total**	11	422

EEA = European Economic Area;

Interview participants were given a letter IA–IK, and survey responders were assigned a number S1–S485 with which they are referenced in the data.

Key themes from the analysis of the responses to the question about the best and worst things about reflection and the interview data are presented.

### Positive aspects of reflection

Table 2 summarises the themes identified describing positive aspects of reflection. Reported in more detail below are four of the most common themes from the data: reflection as an effective learning approach, reflection for self-assessment, reflection for professional development, and developing self-awareness.

**Table 2.** Themes from survey and interview data on positive aspects of reflection

Theme	Survey example	Interview examples
Reflection as an effective learning approach	*'Reflective learning makes you responsible for your own learning and engages you as a clinician. I find it super beneficial.'* (S268)	*'I think learning is the biggest thing about reflection … it’s like revising things … like a problem-solving point of view.'* (ID)
a. Opportunity	*'Gives the clinician the opportunity to step aside from daily practice and think insightfully about events.'* (S94)	*'It gives you an opportunity to reassess your decision making … you will always find room for improvement.'* (IH)
b. Helps	*'Helps me think about how I felt in a situation in hindsight and to learn from my experience.'* (S407)	*'I think it’s a very good exercise, it helps you learn a lot about yourself and the scenario.'* (IK)
c. Consolidate	*'It enables me to consolidate my learning regularly, practically and positively.'* (S344)	*'Sitting down and reflecting on what you have learnt … Putting it on paper … it tends to stay with you longer.'* (ID)
Reflection for self-assessment	*'Reflection allows me to think about what I am doing right or doing wrong. Is there another way I can learn? Why do I feel anxious about this or that and is there another method to improve on?'* (S25)	*'I’ve been finding that it’s a sort of internal dialogue with oneself of “I’m doing good enough, this isn’t good.”'* (IF)
a. Learning needs	*'It gives an opportunity to self-assess and to identify leaning needs.'* (S3)	*'I found that when I was reflective I could identify my gaps and what I needed to learn.'* (IG)
b. Strengths and weaknesses	*'It exposes my deficiencies and strengths which helps direct my learning.'* (S224)	*'I think the purpose would be … appreciate the good things that happened … to identify where your weaknesses are …'* (IG)
Reflection for professional development	*'It helps to objectively analyse scenarios and creates a learning opportunity for better medical practice.'* (S29)	*'You tend to change your practice … it was more effective when I saw a consultant doing it this way so yes, I should apply that next time …'* (IC)
a. Mistakes	*'It helps in improving our knowledge and also preventing repeating mistakes.'* (S250)	*'Each of those learning events I have written I know would not be repeated, because I have learned to be better from them … mistakes you make teach you more.'* (IJ)
Developing self-awareness	*'Helps to know myself and my thinking process better.'* (S376)	*'I’ve learned so much about myself through reflection and I keep asking, why have I acted so …?'* (IE)
a. Emotions	*'It makes me to think of aspects of patient encounters I wouldn't normally think about, like how a case made me feel.'* (S227)	*'I think the best part of the reflection is that it changes you — you stop being a doctor without emotions and you become more human.'* (IA)
Document or record	*'Sometimes its useful to document certain cases as reference for future practice.'* (S295)	*'I feel it also helps you to write it down … because you can go back and have a look at it.'* (II)
Dialogue with trainer	*'I enjoy writing so it can sometimes be fun to put my thoughts, view, feelings etc about situations into words and share that with my trainer.'* (S167)	*'What’s the best thing? It’s to see the comment from my supervisor. I’m like “this is a fun chat.”'* (IG)
Vague	*'Makes you think more.'* (S189)	

#### Reflection as an effective learning approach

Responses in this theme were common in the survey data. Some responses spoke in general terms about the benefits of reflection as a learning method.

*'The best thing about reflection is that you can learn from every case or situation. Once you develop an attitude of regular reflection, you keep learning and improving in your practice.'* (S4)

Within this theme, several sub-themes were apparent. Trainees felt that reflections provided an *opportunity* for learning that may otherwise have been overlooked. The process of reflection also provided some *help* to trainees when thinking through their learning experiences. And finally there was *consolidation* of learning through reflection.

#### Reflection for self-assessment

This was the most common theme among survey responders. Reflection offered an opportunity to take stock, think about things that went well, and consider things that they felt could be improved.

*'Holds one accountable to continue self-assessing and tracking performance deficits and need for personal improvement or to validate and encourage oneself.'* (S279)

Within this theme, there were important sub-themes that were apparent. First, this process *of* self-assessment was noted by many to be a way in which they could *identify learning needs*. Second, for some trainees this self-assessment through reflection enabled them to *identify their strengths and weaknesses*.

#### Reflection for professional development

A further strong theme within the survey data was the notion of reflection for professional development. Rather than taking stock of personal attributes, responses in this theme were specific to clinical encounters.

*'It allows me to go through the case again in retrospect having knowledge of outcomes, so that I can think how I could have managed the same case in better way.'* (S341)

This idea of using experiences to model future consultation was common. Through reflection, trainees spoke of identifying what they would do differently in the future and acknowledged that this was important in improving patient care. Many trainees mentioned mistakes in relation to this; reflection offered an opportunity to learn from mistakes and to prevent them from happening again.

#### Developing self-awareness

Perhaps the most powerful responses, in both the interviews and the survey data, were those that referenced reflection as a means of developing awareness of emotions and of oneself. For some, the best thing about reflection was catharsis; reflection provided a way to deal with difficult situations. For others, reflection was a way of tapping into their feelings.

*'I think the best part of the reflection is that it changes you — you stop being a doctor without emotions and you become more human.'* (IA)

### Negative aspects of reflection

The themes for negative aspects of reflection are summarised in Table 3. The main themes in this area were time, mandatory and forced reflections, and difficult and unfamiliar process. The fear of medico-legal consequences is also reported as it provoked notably strong responses.

**Table 3.** Themes from survey and interview data on negative aspects of reflection

Theme	Survey example	Interview example
Time	*'Having to sit aside and taking time to think and then write … with my kid and family I find it difficult.'* (S297)	*'The time it takes … I think I’m a perfectionist, so it can take me a whole week to write one reflection.'* (IA)
Mandatory or forced	*'The fact that there is a stipulated number of reflection entries in each training period means sometimes the trainee makes entries just to meet up with the required number.'* (S386)	*'It’s just being forced … that restricts you and makes you tailor stuff … it should be more open … we should be able to reflect how we feel a reflection should be.'* (IC)
Difficult and unfamiliar process	*'As we do not have previous reflections, it was very difficult to know what they mean by reflect and how to do this. And when I did that, I am not sure is it the right way or not.'* (S299)	*'When I started ST1 I was always anxious if I‘m saying and writing the right things, if I’m reflecting properly because as I say it’s not something we do.'* (II)
Fear of consequences	*'Difficult to reflect on mistake because of fear of GMC scrutiny or providing evidence for our own weaknesses.'* (S115)	*'I find myself saying’"Okay, delete that, you don’t want that on record". Because some smart lawyer somewhere can twist your words out of context and put you in trouble.'* (IH)
Boring or repetitive	*'Sitting down, recalling the events of a particular case or event, putting on a writer’s hat can be tedious.'* (S5)	*'Sometimes when you’ve had an uneventful week you just think "Okay what do I reflect on?"… everything that we’ve seen, I’ve reflected on it already.'* (ID)
Distressing	*'Sometimes looking back at things and realising things you could have done and you did not makes you feel low, and sometimes that can be overwhelming.'* (S366)	
Documenting	*'We all reflect on the events always, some of us do that in the mind, some do it in writing form. I find it hard to reflect in writing form but does that make me a bad clinician? I don’t think so.'* (S143)	*'We are doing the reflection, but the writing down bit and realising what you have done is the tricky bit for IMGs.'* (IE)
Negative	*'Waste of time.'* (S25)	
Vague	*'Writing too much.'* (S331)	

#### Time

This was by far the most common response recorded in the survey data and was mentioned by several interview participants. There was a sense of reflection adding extra pressure in an already daunting and busy training schedule. Reflections take time to complete and it was difficult to find the time to do them.

*'You don’t get time to do it, but you will know that you’re lagging behind and that’s extra pressure to have on top of your clinical work.'* (ID)

#### Mandatory and forced reflections

The fact that reflections are mandatory for progress through training was a key feature in both interview and survey data. Having a specific number of reflections to be completed was problematic for trainees.

*'Remembering that I have to do a particular number can be stressful and makes it less interesting and the anxiety makes it difficult for me to do it properly.' (*S376)

For many trainees, the fact that reflection was mandated led to a change in their approach to their reflective writing. Both interview and survey participants reported that their entries became forced, that they were tailoring reflective log entries either in an attempt to maintain numbers, or in order to meet certain curriculum requirements.

*'I feel many of my reflections are forced … I have to turn out a couple of reflections just to meet that number. I probably wouldn’t have reflected on them in that way … but I need to tick a box … that’s not you actually reflecting.'* (IK)

There was also evidence that trainees recognised that this formulaic approach felt unnatural and would ultimately devalue the process of reflection.

*'… that restricts you and makes you tailor stuff to that particular criteria … it should be more open … because everyone is different and you don’t have to stick to a certain criteria to be able to reflect …'* (IC)

#### Difficult and unfamiliar process

A clear theme from the survey data was that of reflection as a difficult and unfamiliar process. Trainees mentioned their lack of experience of reflection in undergraduate training, and how writing reflections when English is a second language adds additional challenges.

*'It takes a lot of getting used to. I find it especially challenging because English is not my first language.'* (S96)

#### Fear of repercussions

Although this theme was not as frequently described, the emotive nature of the qualitative responses was notably strong, especially in the interview data. Participants were afraid of their reflective entries being used against them, specifically in a medico-legal context.

*'The worst thing about reflection for me is the fact that I realise I’m putting things down on record … because some smart lawyer somewhere can twist your words out of context and put you in trouble. I find that a major limitation.'* (IH)

Both interview and survey participants referred to the case of Dr Bawa-Garba in this context.

*'I know it’s been years now and the GMC keeps saying it’s not related but the Bawa-Garba case … it has hit me really, really, really hard … what happened, how her reflections and how her portfolio was looked at, it wasn’t great.'* (IE)

*'After the case of Dr Bawa-Garba, it appears certain reflections should be edited.'* (S353)

## Discussion

### Summary

This study analyses the combined free-text responses from national survey data and data from follow-up interviews with IMGs working within UK GP training, to better understand the experiences of reflection for this group of individuals. IMGs described how overall they felt positive about reflection as it provides an effective learning approach, is helpful for self-assessment and personal development, and is a method with which they were able to develop self-awareness.

The most common challenge to reflection was the time taken to complete entries, often compounded by a lack of familiarity with reflection and the challenges of English as a second language. The mandatory aspect of reflection within assessments was a concern; for example, having specific numbered requirements and the pressure to tick off curriculum items made entries feel forced, with many trainees describing ‘*tailoring*’ their entries. There was recognition that this could devalue reflection. It also seems that the effects of the Bawa-Garba case^12^ are still being felt by many IMGs who expressed concerns about confidentiality and fear of medico-legal consequences.

### Strengths and limitations

The analysis in this study triangulated themes from a large sample of qualitative verbatim data from two open questions in a survey and data obtained from purposive in-depth interviews. It is striking that the richness and depth of free-text survey comments mirrored the richness of the verbatim data from the interviews. These two methods of data collection produced the largest qualitative dataset to date on the experience of reflection by IMGs. Our sample of IMGs shows both sex balance and ethnic diversity.

Interview participants were initially recruited from a pool of survey participants who had expressed an interest in further involvement in the research. As trainees self-selected for interview, it is possible strong negative or positive views regarding reflection are over-represented in the data. However, for the purposive second phase of recruitment, the participants had a more general interest in research involvement rather than having specific views on reflection. The large proportion of survey responders who chose to complete optional free-text questions on the best and worst aspects of reflection means the data comes from a wide diversity of participants from across the UK. Considering this diversity, our findings are both credible and transferable.

Interviews were conducted by the lead researcher (LE), who at the time of interviews had recently completed GP training (CCT January 2022). This near-peer approach of data collection, along with the anonymity of transcripts, seems to have allowed trainees to express negative views without fear of repercussions. Participants were also aware that LE was the only person to have access to identifiable data.

In order to maintain reflexivity, field notes were taken, and an iterative approach to data collection and analysis was maintained through regular team meetings (LE, BJ, CM), with findings subjected to critical interpretive challenge. Self-awareness about disciplinary bias was crucial during analysis as BJ and CM are academic GPs who are also GP trainers with experience of supervising IMGs. A stakeholder group comprising doctors with experience of working and training abroad was involved at all stages of the research including development of the interview schedule and analysis of data.

### Comparison with existing literature

According to the AMEE guidance for reflection in medical education, reflection is key to self-regulated life-long learning and the development of professional expertise. This is because it enables greater understanding of the self and situation that then informs future actions.^13^ Certainly there is considerable overlap with the themes from our data, which suggest that IMGs see the potential for professional development through assessment of the self and clinical situation as key benefits of reflection.

IMGs are known to have less experience of reflection as a learning approach in their undergraduate training than their UK-trained colleagues,^9^ and it has been suggested that early introduction to and understanding of reflection could be beneficial to their progress in training.^14^ The data presented here suggest that although reflection may be unfamiliar for IMGs, they have a good understanding of the benefits of reflection in respect to GP training. Difficulties encountered in engaging with reflection are unlikely to be related to a lack of understanding of these benefits, more so that reflection is time-consuming, especially when reflection itself is unfamiliar and required to be submitted in writing when English may be a second language.

It is, however, important to remember that this group is by no means homogenous; individual trainees will each have different experiences of education. Indeed, the term IMG simply relates to people who have obtained their medical qualification in a country other than the UK. Some may have been educated to secondary school level in the UK before choosing to complete their medical degree abroad, others may have extensive experience of the NHS through foundation training and previous specialty posts before embarking on GP training.

Previous literature has made clear that when reflective writing is submitted as a form of assessment, this changes the nature of what is written; students write what they think their assessors want to hear, rather than a true account of their experiences.^2–4^ Our study suggests this is the case for many trainees, that having curriculum competencies that they need to evidence, and numbers they need to fulfil, means that entries are perceived as forced. Some change what they write depending on who will read it. It could be argued that this negates the benefits of reflection as a tool of personal and professional development entirely, instead creating the ‘*reflective zombies’* described by de la Croix and Veen.^15^

Concerns about confidentiality of reflective entries and fear of medico-legal consequences seem related to the effects of the Bawa-Garba case;^12^ specifically reports in the media that the doctor’s reflections from their online portfolio were used as evidence against them in court.^16^ Several trainees made direct reference to this case in their responses. This correlates with the findings from a previous study in which GP trainees’ engagement in reflection post-Bawa-Garba showed considerable anxiety about confidentiality of entries, with international graduates in particular being identified as more vulnerable to the medico-legal consequences of reflection owing to their lack of familiarity with this method of learning.^10^

### Implications for research and practice

This mixed-methods study provides important insight into IMGs’ views of reflection as part of GP training. Although most IMGs are unlikely to have experienced reflection as part of their undergraduate training, our study suggests that they are aware of the benefits of reflection for personal and professional development.

In order for IMGs to engage effectively in reflection, we need to support them in the development of this important skill, taking into account some of the difficulties highlighted in this research. We would encourage GP training educators to focus on quality rather than quantity when making their recommendations for number of written reflections expected of trainees in their portfolios. From interview data, requirements varied across the UK; for example, numbers of written reflections expected ranged from one entry every fortnight to 3–4 per week. Mandating high numbers of reflections from trainees is likely to contribute to the sense of entries feeling forced, adding to time pressure and potentially limiting learning from reflection.

On an individual level, GP educators are encouraged to engage in a discussion with their trainees about their experiences of reflection and their confidence in written reflections. The data show that within this group of individuals there is diversity in experience and enjoyment of written reflection. Where there is less experience of reflection, trainers may find that engaging in a reflective conversation with trainees will assist in their understanding, allowing trainees to be guided through a case or experience with questions and feedback.

Medico-legal concerns continue to be a main barrier to engaging with reflection. To address this, we suggest approaching reflection from a more theoretical perspective as described by Moon.^17^ The focus on reflection from a theoretical perspective is on the reaction to an event rather than the description of it, and is therefore less problematic from a medico-legal viewpoint. Several local schemes in the Yorkshire and the Humber GP school have adopted Moon’s exercise ‘The Park’^18^ in their reflective training, and LE has produced a series of 3–4-minute videos and short exercises to guide reflection according to Moon’s principles. We would recommend that further research aims to identify and evaluate such resources for supporting reflection so that recommendations for best practice can be made and adopted nationally.

Ultimately, we would suggest that the RCGP considers its use of reflection as a means to evidence curriculum coverage carefully, because by using reflection in this way, we risk devaluing reflection as a tool for professional development. In the meantime, we hope these evidence-based practical tips will be adopted by GP trainers to help their IMGs overcome the challenges presented by reflection.

## Data

The dataset relied on in this article is available from the corresponding author on reasonable request.

## References

[1] Royal College of General Practitioners (RCGP) (2023). WPBA learning log.

[2] Ross J (2011). Traces of self: online reflective practices and performances in higher education. Teaching in Higher Education.

[3] Hargreaves J (2004). So how do you feel about that? Assessing reflective practice. Nurse Educ Today.

[4] Hobbs V (2007). Faking it or hating it: can reflective practice be forced?. Reflective Practice.

[5] Mann K, Gordon J, MacLeod A (2009). Reflection and reflective practice in health professions education: a systematic review. Adv Health Sci Educ Theory Pract.

[6] General Medical Council (GMC) (2018). Outcomes for graduates.

[7] Khan FA, Chikkatagaiah S, Shafiullah M (2015). International medical graduates (IMGs) in the UK—a systematic review of their acculturation and adaptation. J Int Migr Integr.

[8] RCGP (2023). Fit for the future: opening the door to international GPs.

[9] Emery L, Jackson B, Oliver P, Mitchell C (2022). International graduates’ experiences of reflection in postgraduate training: a cross-sectional survey. BJGP Open.

[10] Emery L, Jackson B, Herrick T (2021). Trainee engagement with reflection in online portfolios: a qualitative study highlighting the impact of the Bawa-Garba case on professional development. Med Teach.

[11] Braun V, Clarke V (2006). Using thematic analysis in psychology. Qual Res Psychol.

[12] Pulse (2019). Bawa-Garba: Timeline of a case that has rocked medicine.

[13] Sandars J (2009). The use of reflection in medical education: AMEE Guide No. 44. Med Teach.

[14] Warwick C (2014). How international medical graduates view their learning needs for UK GP training. Educ Prim Care.

[15] de la Croix A, Veen M (2018). The reflective zombie: problematizing the conceptual framework of reflection in medical education. Perspect Med Educ.

[16] Cohen D (2017). Back to blame: the Bawa-Garba case and the patient safety agenda. BMJ.

[17] Moon J (2007). Getting the measure of reflection: considering matters of definition and depth. J Radiother Pract.

[18] Moon JA (2004). A handbook of reflective and experiential learning: theory and practice.

